# Age-related decline in melatonin contributes to enhanced osteoclastogenesis via disruption of redox homeostasis

**DOI:** 10.1186/s10020-024-00779-x

**Published:** 2024-01-12

**Authors:** Di-Zheng Wu, Guo-Zheng Zhu, Kai Zhao, Jia-Wen Gao, Gui-Xing Cai, Hong-Zhou Li, Yu-Sheng Huang, Chen Tu, Jing-Shen Zhuang, Zhi-Wei Huang, Zhao-Ming Zhong

**Affiliations:** 1 Division of Spine Surgery, Department of Orthopaedics, Nanfang Hospital, Southern Medical University, 1838 North Guangzhou Avenue, Guangzhou, 510515 China; 2 Department of Orthopaedics, First Affiliated Hospital of Gannan Medical University, Ganzhou, 341000 China

**Keywords:** Aging, Bone loss, Melatonin, Osteoclastogenesis, Osteoporosis, Oxidative stress

## Abstract

**Background:**

Increased oxidative stress contributes to enhanced osteoclastogenesis and age-related bone loss. Melatonin (MT) is an endogenous antioxidant and declines with aging. However, it was unclear whether the decline of MT was involved in the enhanced osteoclastogenesis during the aging process.

**Methods:**

The plasma level of MT, oxidative stress status, bone mass, the number of bone marrow-derived monocytes (BMMs) and its osteoclastogenesis were analyzed in young (3-month old) and old (18-month old) mice (n = 6 per group). In vitro, BMMs isolated from aged mice were treated with or without MT, followed by detecting the change of osteoclastogenesis and intracellular reactive oxygen species (ROS) level. Furthermore, old mice were treated with MT for 2 months to investigate the therapeutic effect.

**Results:**

The plasma level of MT was markedly lower in aged mice compared with young mice. Age-related decline in MT was accompanied by enhanced oxidative stress, osteoclastogenic potential and bone loss. MT intervention significantly suppressed the receptor activator of nuclear factor-κB ligand (RANKL)-induced osteoclastogenesis, decreased intracellular ROS and enhanced antioxidant capacity of BMMs from aged mice. MT supplementation significantly attenuated oxidative stress, osteoclastogenesis, bone loss and deterioration of bone microstructure in aged mice.

**Conclusions:**

These results suggest that age-related decline of MT enhanced osteoclastogenesis via disruption of redox homeostasis. MT may serve as a key regulator in osteoclastogenesis and bone homeostasis, thereby highlighting its potential as a preventive agent for age-related bone loss.

**Supplementary Information:**

The online version contains supplementary material available at 10.1186/s10020-024-00779-x.

## Introduction

Bone mass is maintained by continuous remodeling through a coupled process of bone resorption and bone formation (Compston et al. 2019). With age, bone resorption slowly begins to exceed new bone formation, which causes gradual loss of bone mass and deterioration of bone microstructure, leading ultimately to the development of osteoporosis (Cui et al. 2022). Bone resorption is carried out by osteoclasts, which are multinucleated cells formed by fusion of monocyte/macrophage precursors. The formation of osteoclasts, also called osteoclastogenesis, is a multi-stage process, including cell commitment, cell–cell fusion, and maturation (Moller et al. 2020; Jevon et al. 2002). Osteoclastogenesis is highly regulated by various endogenous and exogenous molecules, including reactive oxygen species (ROS) (Lee et al. 2005; Li et al. 2021). Aging is generally accompanied by the excess accumulation of ROS and oxidative stress, which contribute to enhanced osteoclastogenesis and bone loss (Manolagas. 2010). It is well established that scavenging ROS and alleviating oxidative stress are beneficial for suppressing of osteoclastogenesis and alleviating age-related bone loss (Zhuang et al. 2021; Liu et al. 2019).

Melatonin (MT) is a neurohormone secreted mainly by the pineal gland with the highest levels during the dark phase and negligible levels during the light phase (Li et al. 2019). Apart from the well-known circadian rhythms regulation, MT serves as a most potent endogenous antioxidant and plays a pivotal role in maintaining redox homeostasis (Galano et al. 2018; Rodriguez et al. 2004; Veneroso et al. 2009; Peyrot et al. 2008; Deng et al. 2006). Peak level of MT declines with aging and coincides with the age-related impairment of antioxidant potential (Rasmussen et al. 2001). It is well demonstrated that MT supplementation can improve antioxidant defense functions in both human and experimental animals (Kedziora-Kornatowska et al. 2008; Chen et al. 2020). Evidence has also suggested that MT may influence skeletal growth and bone formation (Li et al. 2019). MT treatment at pharmacological doses increases bone mass by increased bone formation and reduced bone resorption in vivo (Satomura et al. 2007; Munmun et al. 2021), and suppresses osteoclastogenesis through the attenuation of intracellular ROS in vitro (Zhou et al. 2017). Therefore, it is important to understand the role of MT as an antioxidant in regulating bone homeostasis.

Previous studies have demonstrated that reduced MT secretion is associated with many age-related diseases and pathological situations (Fishbein et al. 2021). The decline of MT may contribute to enhanced osteoclastogenesis and age-related bone loss, but this possibility hasn’t been thoroughly investigated. Here, we reported that age-related decline of MT is associated with increased oxidative stress, osteoclastogenesis and bone loss, and MT can inhibit osteoclastogenesis by enhanced antioxidant capacity both in vivo and in vitro. MT supplementation may provide potential benefits for age-related bone loss.

## Materials and methods

### Animals, reagents, and antibodies

All animals were purchased from the Southern Medical University Experimental Animal Centre (Guangzhou, China). Animal procedures were conducted in accordance with the National Institutes of Health guidelines for the care and use of experimental animals and were approved by the Southern Medical University Institutional Animal Care and Use Committee. The 18-month-old C57BL/6 mice were used as an animal model of age-related osteoporosis (Shao et al. 2022). Young mice (3 month-old) were used as controls. In some experiments, the aged mice were injected intraperitoneally with or without MT (50 mg/kg/day) for 2 months (Khan et al. 2017; Ma et al. 2020). There were 6 mice in each group. Experimental mice were housed under the standard specific pathogen-free conditions with a 12 h light/12 h dark cycle at 20 ± 2 °C. After 24 h of the last MT injection, the mice were euthanized, blood samples were collected in a centrifuge tube with anticoagulant (heparin) at 10:00–11:00 AM, and the plasma was isolated and examined using ELISAs. After the femurs were dissected, some of them were frozen in liquid nitrogen for protein extractions. The remaining femur were stabilized in 4% paraformaldehyde for subsequent micro-computed tomography (CT) scanning, and tartrate-resistant acid phosphatase (TRAP) staining.

Receptor activator of nuclear factorκB ligand (RANKL) and macrophage colony stimulating factor (M-CSF) were purchased from PeproTech (Rocky Hill, NJ, USA). Fetal bovine serum (FBS), alpha minimum essential medium (α-MEM), penicillin, streptomycin, trypsin-ethylenediaminetetraacetic acid (Trypsin/EDTA), 4′,6-diamidino-2-phenylindole (DAPI), protease inhibitor tablets, horseradish peroxidase-conjugated secondary antibodies, SuperSignal West Pico Substrate, and CLXPosure Film were purchased from Gibco Thermo Fisher Scientific (Waltham, MA, USA). MT, dimethyl sulfoxide (DMSO), phosphate buffered saline (PBS), paraformaldehyde, bovine serum albumin (BSA), Triton X-100, and 2′,7′-dichlorofluorescein diacetate (DCFH2-DA) were obtained from Sigma-Aldrich (St. Louis, MO, USA). Stock solution of MT was prepared by dissolving in DMSO to the concentration of 500 mM and diluting in α-MEM to different concentrations as specified in individual experiments.

### Isolation and culture of primary bone marrow-derived monocytes (BMMs)

Primary BMMs were isolated from the femurs and tibias of C57BL/6 mice. After dissecting aseptically under a laminar airflow hood, the ends of the bones were cut off with scissors and the marrow cells were flushed out using a sterile needle and syringe containing α-MEM. Red blood cells were removed by treatment with red blood cell lysis buffer (Beyotime Institute of Biotechnology, Haimen, China). After washing, the cells were cultured in α-MEM containing 10% FBS, 100 U/mL penicillin, and 100 μg/mL streptomycin at 37 °C with 5% CO_2_ in 60-mm cell culture dishes. After incubating overnight, the cell suspension was collected and re-cultured in a complete medium supplemented with 100 ng/mL M-CSF. After a 3-day culture, non-adherent cells were removed by washing with PBS and the adherent cells were cultured until they reached 90% confluence. In subsequent experiments, BMMs were counted and seeded at 5000 cells per well in 96-well plates and 10,000 cells per well in 48-well plates. To observe cell morphology, bright field images of monocytes were collected using a Zeiss Axiovert 40 CFL phase contrast microscope (Carl Zeiss Ltd., Oberkochen, Germany).

### Assay of cell proliferation

Cell proliferation of BMMs was evaluated using the Cell Counting Kit-8 (CCK-8; Beyotime). Ten microliters of the CCK-8 solution were added to each well in 96-well plates and cells were incubated for 1 h at 37 °C. Absorbance was determined at 450 nm using a microplate spectrophotometer (BioTek, Winooski, VT, USA).

### In vitro osteoclast differentiation

BMMs were induced toward osteoclasts by plating 5 × 10^4^ cells per well in a 24-well tissue culture plate and incubating with osteoclast differentiation media (α-MEM containing 10% FBS, 100 U/mL penicillin, 100 μg/mL streptomycin, 20 ng/mL M-CSF, and 50 ng/mL RANKL). Cells were cultured for 5 days with a change of culture media and cytokine supplementation every other day.

### Bone resorption assay

In vitro, BMMs were seeded on bovine cortical bone slices (6 × 6 mm size and 0.2 mm thickness) and were treated as described above. After 6 days, adherent cells were removed and the bone slices were scanning electron microscopy (Hitachi S-3000N, Japan) to observe the bone resorption pits.

### Flow cytometry and cell sorting

Cells were simultaneously stained for CD11b (PerCP-Cy5.5-conj., cloneM1/70), Ly6-G (Brilliant Violet650-conj., cloneRB6-8C5, all eBioscience) and Ly6-C (eFluor-conj., cloneAL-21). BMMs were characterized as CD11b^+^Ly6C^+^Ly6G^−^. For flow-cytometric analyses, we used a BD FACSCanto II (Becton Dickinson Immunocytometry Systems, San Jose, CA, USA). For cell sorting, Monocytes from mice were stained with CD11b, Ly6C and Ly6G antibodies and then isolated using a BD FACSAria II sorter.

### Micro-CT scanning

The bilateral femur was fixed with 4% PFA for 24 h and then scanned with a Micro-CT system (μCT80, SCANCO MEDICAL, Switzerland) as in our previous research (Zhu et al. 2018). Bone mineral density (BMD), bone volume over total volume (BV/TV), trabecular thickness (Tb/Vt.Th), trabecular number (Tb/Vt.N) and trabecular spacing (Tb/Vt.Sp), were calculated using the CTAn software.

### Measurement of intracellular reactive oxygen species (ROS)

Intracellular levels of ROS were quantified by using a 2′,7′-dichlorofluorescein diacetate (DCFH2-DA) method. Monocytes were washed once with PBS and then incubated in α-MEM containing 50 μM DCFH2-DA at 37 °C for 30 min. After washing, cells were treated with MT at 100 μM. To investigate the role of ROS in osteoclastogenesis, cells were incubated in osteoclastogenic differentiation media supplemented with 100 μM MT. Fluorescence of the samples was measured at 488 nm excitation/525 nm emission by a SynergyMx multimode microplate reader (BioTek).

### Tartrate-resistant acid phosphatase (TRAP) staining

Osteoclasts were washed twice with PBS, fixed with 4% paraformaldehyde for 15 min, and stained with a Leukocyte Acid Phosphatase Kit (Sigma-Aldrich) according to the manufacturer’s instructions. After rinsing with deionized water, the fixed cells were incubated with Fast Garnet GBC Base solution (7.0 mg/mL in 0.4 mol/L hydrochloric acid) and sodium nitrite solution (0.1 mol/L) for 2 min. The cells were then stained with a mixture of naphthol AS-BI phosphate solution (12.5 mg/mL) and tartrate solution (0.335 mol/L, pH = 4.9 ± 0.1) in acetate buffer (2.5 mol/L, pH = 5.2 ± 0.1) in the dark for 10 min at 37 °C. The nuclei were counterstained with DAPI. Digital images of red-stained osteoclasts were captured by an Olympus IX51 microscope (Olympus Corporation, Tokyo, Japan). Multinucleated TRAP-positive cells with more than three nuclei were counted as osteoclasts. The number of TRAP-positive osteoclasts and the number of nuclei within these osteoclasts were counted in ten randomly chosen fields of view (FOV).

### Western blot analysis

Cells were dissolved in ice-cold cell lysis buffer (Beyotime) containing protease inhibitors; the protein concentration in cell extracts was quantified using a BCA protein assay kit (Beyotime). Equal amounts of protein from each extract were denatured and separated in a 10% polyacrylamide gel (Beyotime) and transferred by electrophoresis onto a nitrocellulose membrane (Thermo Fisher Scientific). The membrane was incubated with diluted primary antibodies against TRAP (#32694, 1:1000, SAB), cathepsin K(CTSK) (11239-1-AP, 1:1000, Proteintech), c-Fos (66590-1-Ig, 1:1000, Proteintech), NFATc1 (66963-1-Ig, 1:2000, Proteintech), NF-кB p65 (10745-1-AP, 1:1000, Proteintech), phospho-p65(ab76302, 1:1000, Abcam), IкB-α (51066-1-AP, 1:1000, Proteintech), phospho-IкB-α(ab133462, 1:10000, Abcam), Nrf2 (16396-1-AP, 1:5000, Proteintech), Keap1 (10503-2-AP, 1:2000, Proteintech), HO1 (10701-1-AP, 1:1000, Proteintech), or GAPDH (10494-1-AP, 1:10000, Proteintech) at 4 °C overnight, followed by the secondary antibody of horseradish peroxidase-conjugated goat anti-mouse or anti-rabbit for 1 h at room temperature. SuperSignal West Pico Substrate and CLXPosure Film were used for exposure. The intensity of the bands was quantified using the ImageJ software.

### Immunofluorescence staining

An immunofluorescence staining was used to determine the effects of MT on the expression of Nrf2 and the nuclear translocation of P65. The control group and MT-treated monocytes were fixed with 4% paraformaldehyde for 15 min. Then permeabilized the cells with 0.3% Triton X‐100 for 5 min and blocked with 3% BSA in PBS. The cells were incubated with anti-P65 or anti-Nrf-2 antibody followed by biotinylated goat anti-rabbit IgG antibody and fluoresce in conjugated streptavidin (Vector Laboratories, CA, USA).

### Quantitative real-time polymerase chain reaction (qRT-PCR)

Total RNA was extracted using the TRIzol^®^ reagent (Thermo Fisher Scientific) and reversely transcribed to complementary DNA (cDNA) by the RevertAid FirstStrand cDNA Synthesis Kit (Thermo Fisher Scientific). Quantitative real-time reverse transcription-polymerase chain reaction (qRT-PCR) was performed on a CFX96™ Real-Time PCR System (Bio-Rad) using the iTap™ Universal SYBR^®^ Green Supermix kit (Bio-Rad, Hercules, CA, USA) according to the manufacturer’s protocol. Relative transcript levels of target genes were calculated using the comparative Ct (2 − ΔΔCt) method and expressed as a fold change respective to the control. The primer sequences are listed in Table 1.

**Table 1:** The sequences of primers used for qRT-PCR

GAPDH-F	AGGTCGGTGTGAACGGATTTG
GAPDH-R	TGTAGACCATGTAGTTGAGGTCA
TRAP-F	ACTTGCGACCATTGTTAGCC
TRAP-R	AGAGGGATCCATGAAGTTGC
CTSK-F	AGCAGAACGGAGGCATTGACTC
CTSK-R	TTTAGCTGCCTTTGCCGTGGC

### Enzyme-linked immunosorbent assay (ELISA)

Each blood sample was centrifuged at 3000 rpm and 4 °C for 15 min and the plasma supernatant was removed and placed in a fresh centrifuge tube. The levels of melatonin (MT), advanced oxidative protein products (AOPPs), malondialdehyde (MDA) and superoxide dismutase-1 (SOD-1) were quantified using the respective ELISA kits, in accordance with the manufacturer’s instructions. Absorbance at 450 nm was measured using a microplate reader (S/N 415-2687, Omega Bio-Tek, Ortenberg, Germany).

### Statistical analysis

Statistical analysis was conducted using the SPSS 13.0 statistical software (SPSS Inc., Chicago, IL, USA). All data were expressed as means ± standard deviation (S.D.). The Shapiro–Wilk test was used to analyze the normality and equal distribution of variance between the different groups. For data with normal distribution, statistical analyses were performed with Student’s t-test or ANOVA analyses of variance unless otherwise stated. Significance was indicated by a p-value.

## Results

### Decline of MT was accompanied by the enhanced osteoclastogenic potential and oxidative stress in the aging process

**Fig. 1:**
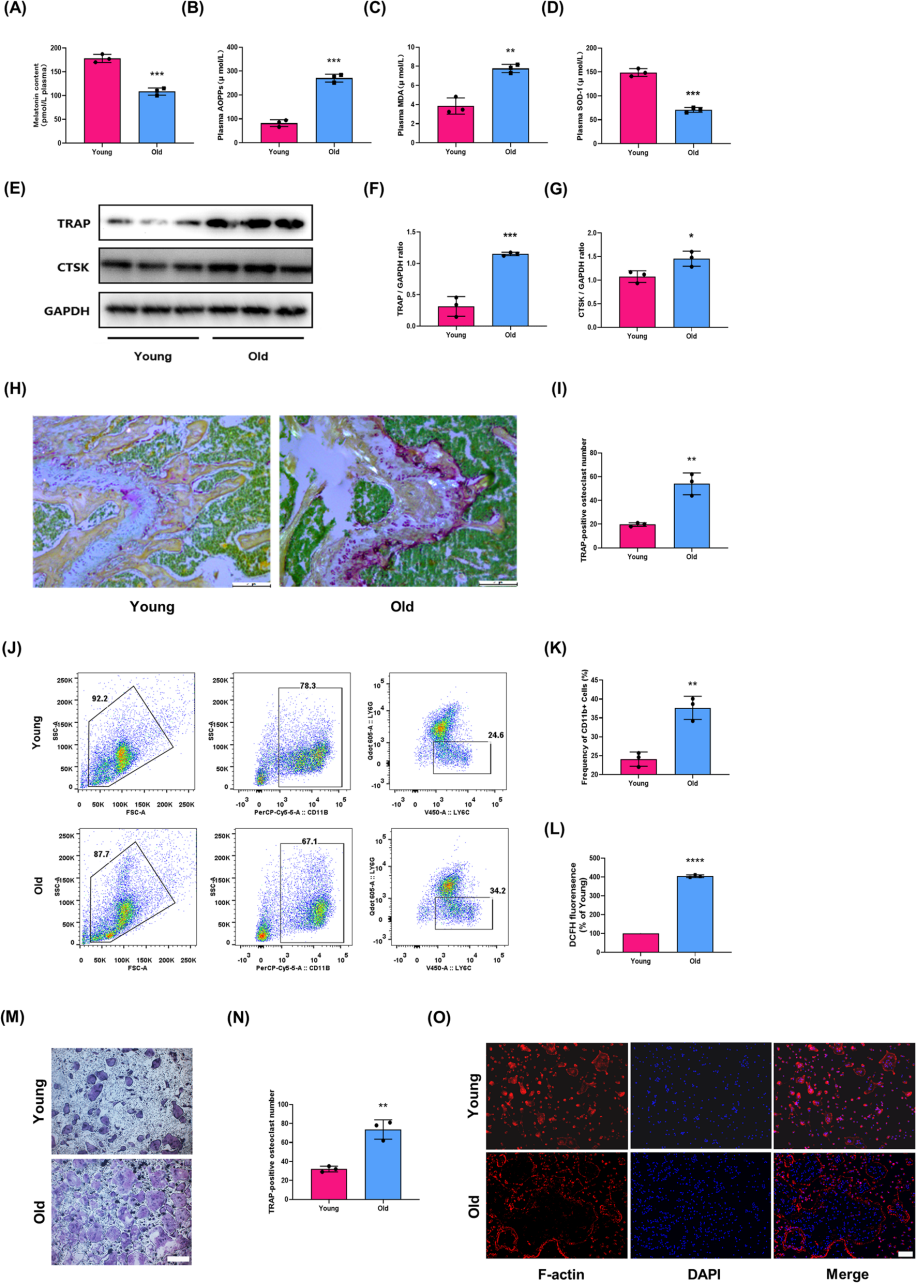
The decline of melatonin (MT) was accompanied by the enhanced osteoclastogenic potential and oxidative stress in the aging process. **A**–**D** The plasma concentration of MT, oxidative stress markers including AOPPs, MDA and SOD-1 in young(3-month-old) mice and old (18-month-old) mice. Data represent mean ± S.D. of at least three independent experiments (n = 3 per group). **E**–**G** Western blot analysis of the TRAP and CTSK expression in the femoral metaphysis. **H**–**I** Representative images of TRAP staining for assessment of the number of TRAP-positive cells in the femoral metaphysis. Scale bar = 200 μm. **J**–**K** Flow cytometry analyses of the number and proportion of monocytes in the bone marrow cavity. Data represent mean ± S.D. of at least three independent experiments (n = 6 per group). **L** DCFH fluorescence analyses of intracellular ROS level in bone marrow monocytes (BMMs) from young and old mice. Data represent mean ± S.D. of at least three independent experiments (n = 3 per group). (M) Representative TRAP staining images of osteoclast differentiation of BMMs from young and old mice. Scale bar = 100 μm. **N** Quantification of TRAP + multinucleated cells (> 3 nuclei/cell) per field. **O** Representative rhodamine's Phalloidin staining for F-actin ring formation in BMMs from young and old mice. Scale bar = 50 μm. *p < 0.05, **p < 0.01, ***p < 0.001, ****p < 0.0001

**Fig. 2:**
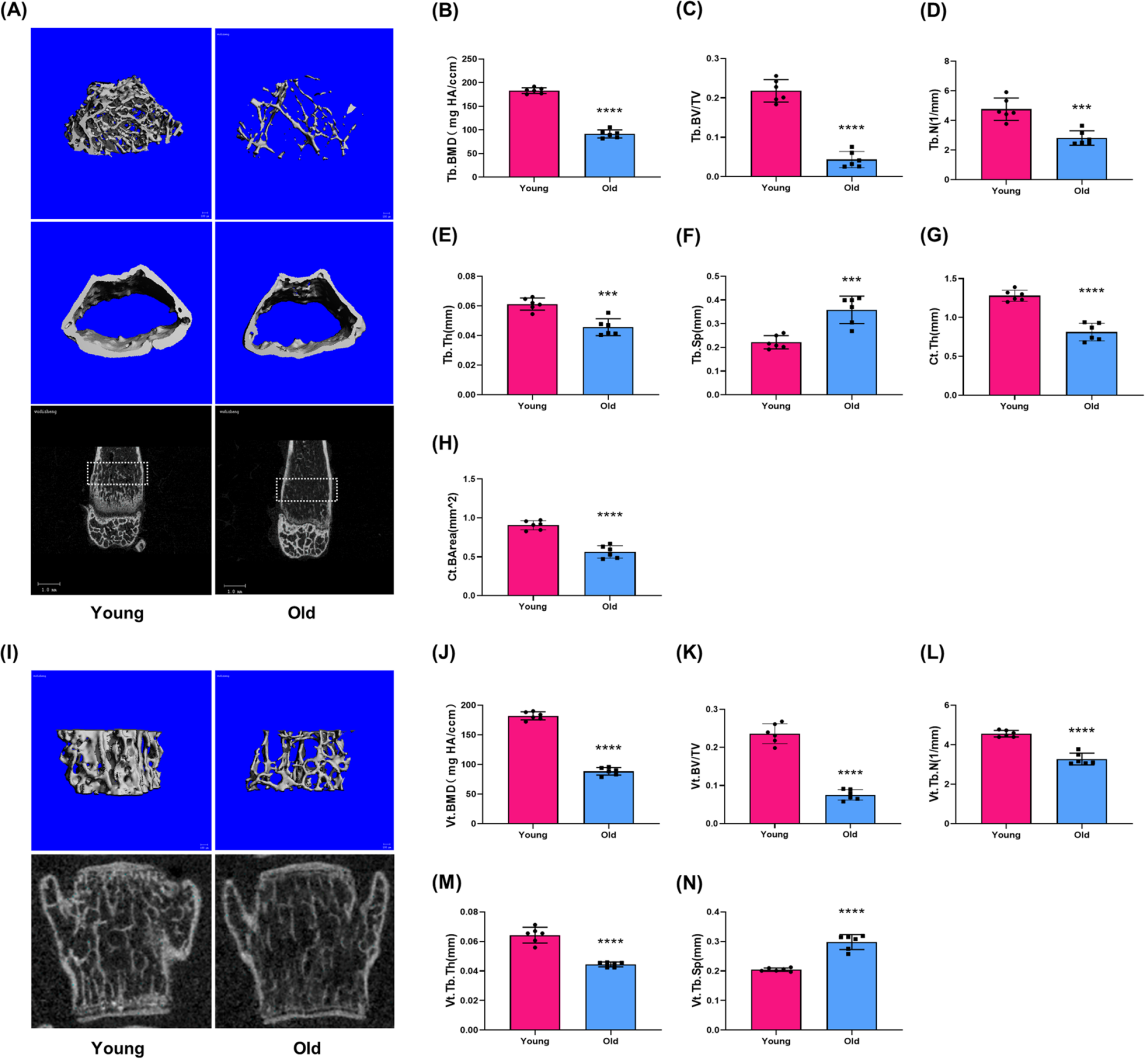
Aged mice exhibited bone loss and deterioration of bone microstructure. **A** Representative micro-CT images of the distal femur from young (3-month-old) mice and old (18-month-old) mice. **B**–**H** Quantitative parameters in the distal femur, including bone mineral density (BMD), bone volume fraction (BV/TV), trabecular thickness (Tb/Vt.Th), trabecular number (Tb/Vt.N), trabecular separation (Tb/Vt.Sp), cortical bone thickness (Ct.th) and average cortical bone area (Ct.BArea). (I) Representative μCT images of L4 vertebral bodies. **J**–**N** Quantitative parameters in L4 vertebral bodies. Data represent mean ± S.D. of at least three independent experiments (n = 6 per group). ***p < 0.001; ****p < 0.0001

To address the age-related changes in MT level and osteoclastogenesis, the plasma levels of MT and oxidative stress markers were measured, the osteoclastogenic potential and bone microstructure were also detected. When compared with young mice, aged mice exhibited a sharp decline in the plasma level of MT (Fig. 1A), corresponding to a significant increase in oxidative stress markers, such as AOPPs, MDA (Fig. 1B, C) and a decrease in SOD-1(Fig. 1D). The expression of bone resorption markers, TRAP and CTSK, were higher in femoral metaphysis of aged mice than that in young mice (Fig. 1E–G). A significantly higher number of TRAP-positive multinucleated cells was shown in the femoral metaphysis from aged mice compared with the young mice (Fig. 1H, I). A marked increase in the number of monocytes in the bone marrow cavity was also observed in aged mice (Fig. 1J, K). Furthermore, in vitro assay showed that the level of intracellular ROS was significantly higher in BMMs from aged mice than that in young mice (Fig. 1L). The BMMs from aged mice also showed a higher capacity of differentiation into TRAP-positive multinucleated cells (Fig. 1M, N) and F-actin ring formation (Fig. 1O). Moreover, compared with young mice, aged mice showed a loss of bone mass, deterioration of bone microstructure and cortical bone thinning in the aging process (Fig. 2). The above data indicated that a decline of MT level was associated with the enhanced osteoclastogenesis and oxidative stress in the aging process.

### MT suppressed osteoclastogenesis of BMMs from aged mice

**Fig. 3:**
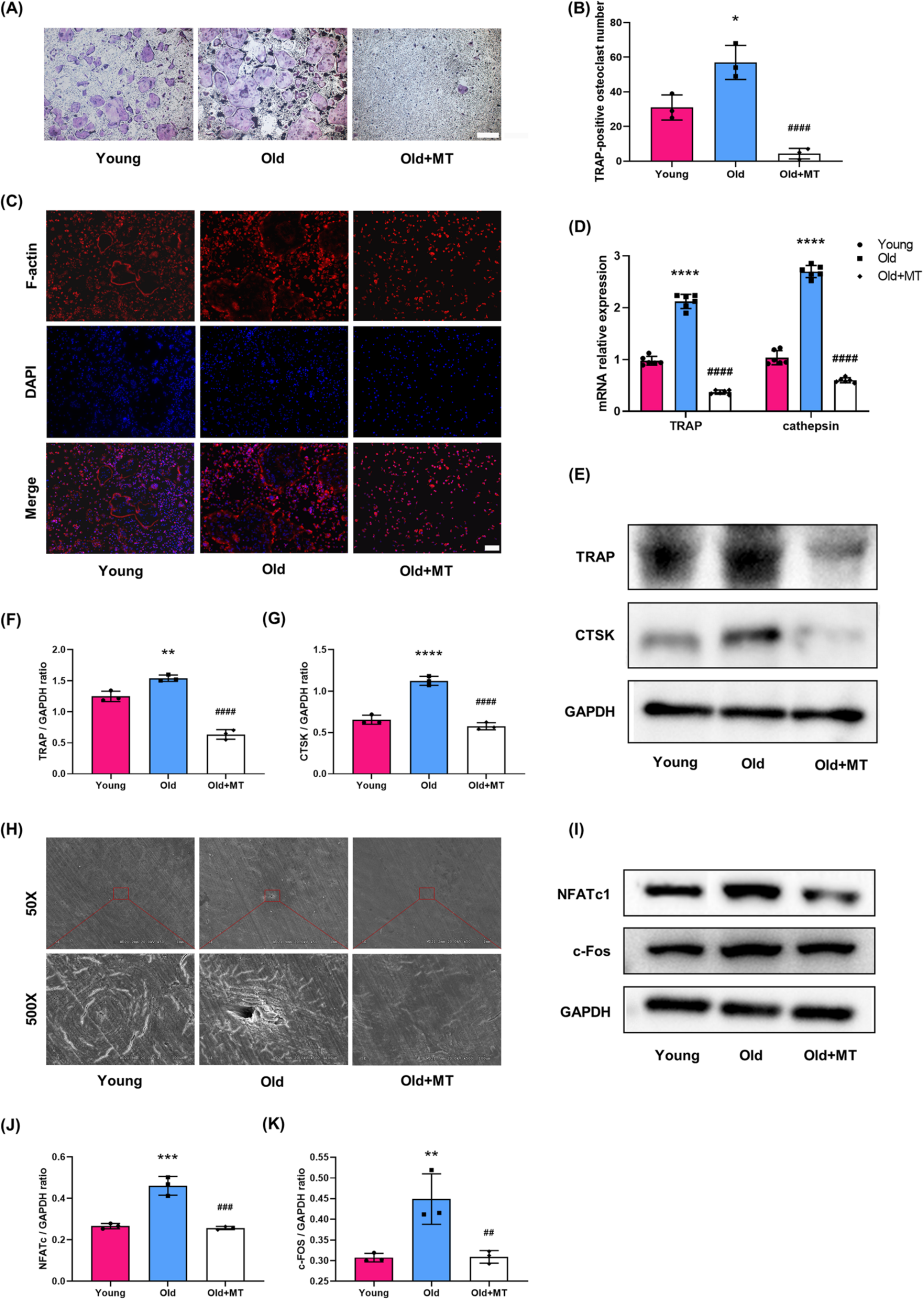
MT suppressed osteoclastogenesis of BMMs from aged mice. **A** Representative TRAP staining images of osteoclast differentiation of BMMs from young mice, old mice and old mice with MT treatment. Scale bar = 100 μm. **B** Quantification of TRAP + multinucleated cells (> 3 nuclei/cell) per field. **C** Representative rhodamine's Phalloidin staining for F-actin ring formation in BMMs from three groups. Scale bar = 50 μm. **D** qRT-PCR analyses of the gene expression of TRAP and CTSK. (E–G) Western blot analysis of protein levels of TRAP and CTSK. (H) Representative electron microscop scanning for the formation of bone resorption pits. **I**–**K** Western blot analysis of protein levels of osteoclast-related transcription factors, c-Fos and NFATc1. *p < 0.05, **p < 0.01, ***p < 0.001, ****p < 0.0001 compared with the young group; ^##^p < 0.01, ^###^p < 0.001, ^####^p < 0.0001 compared with the old group

**Fig. 4:**
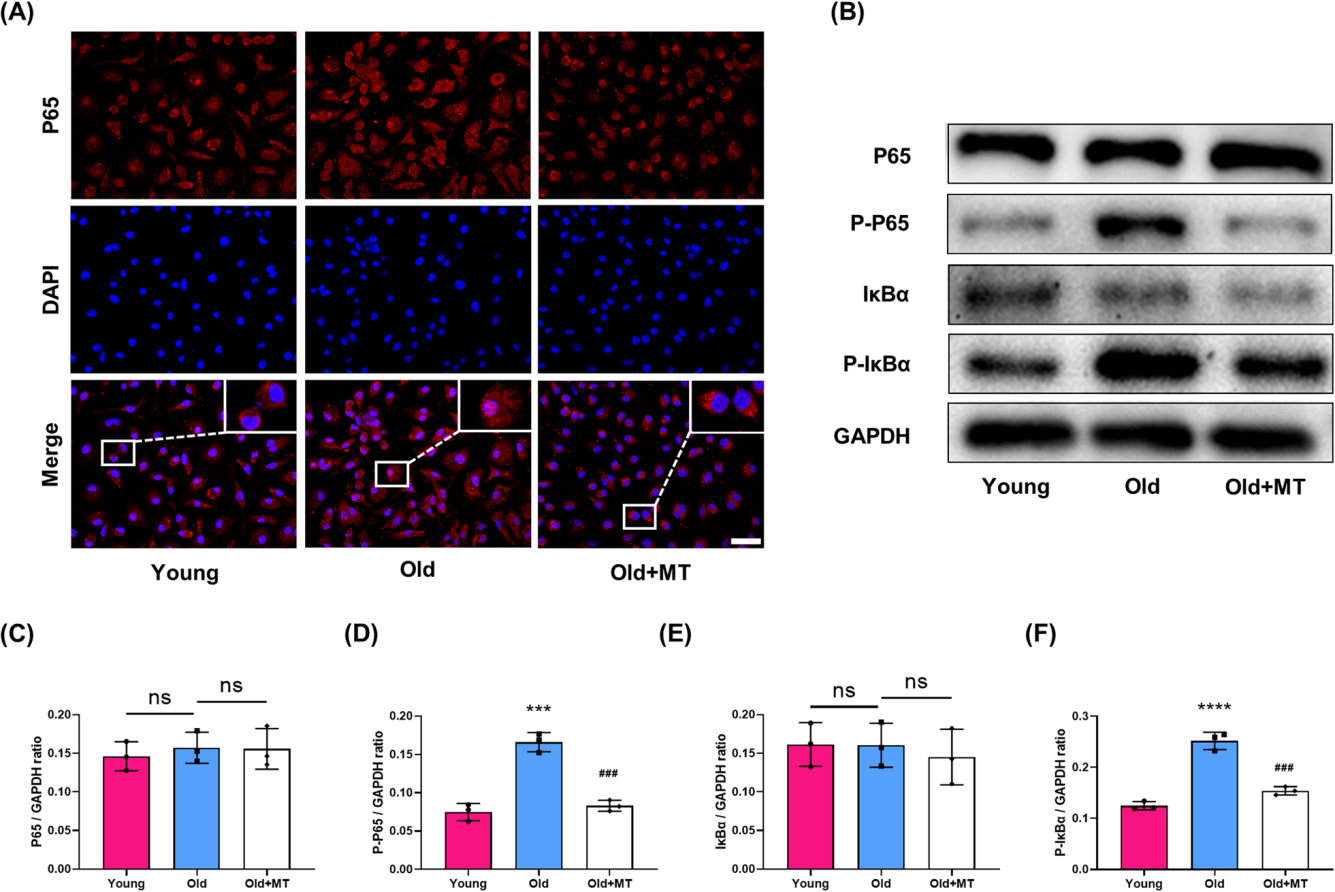
MT inhibited phosphorylation of IκB and p65 in BMMs from aged mice. **A** Representative fluorescence images of p65 translocation from the cytoplasm to the nucleus. **B**–**F** Western blot analysis of protein levels of p65, P-p65, IκBα, P-IκBα. ***p < 0.001, ****p < 0.0001 compared with the young group; ^###^p < 0.001 compared with the old group; ns, not significant

In order to investigate the effects of MT on osteoclastogenesis in vitro, BMMs from aged mice were cultured in the presence of M-CSF and RANKL with or without MT. First, we found that MT of 100 μM had an inhibitory effect on intracellular ROS production and osteoclastogenesis, but no significant effect on cell proliferation, and was therefore used as the optimal drug concentration (Additional file 1: Fig. S1). MT treatment significantly decreased TRAP positive multinuclear cells (Fig. 3A, B) and inhibited the formation of F-actin ring (Fig. 3C). MT treatment also inhibited the gene and protein expression of osteoclast differentiation markers, such as TRAP and CTSK (Fig. 3D–G). The bone resorption pits assay showed that MT treatment significantly inhibited the bone resorption activity of osteoclasts (Fig. 3H). Previous studies showed that NFATc1, c-Fos and NF-κB were considered important regulators regulators for osteoclastogenesis (Kim et al. 2014; Ni et al. 2020). MT treatment also inhibited the expression of c-fos and NFATc1 expression (Fig. 3I–K). As shown in Fig. 4A–F, following RANKL stimulation, phosphorylation of IκB and p65 was enhanced in BMMs from aged mice compared to young mice, and translocation of p65 from the cytoplasm to the nucleus was also increased, Importantly, this above process was inhibited by MT treatment.

### MT enhanced antioxidant capacity of BMMs from aged mice

**Fig. 5:**
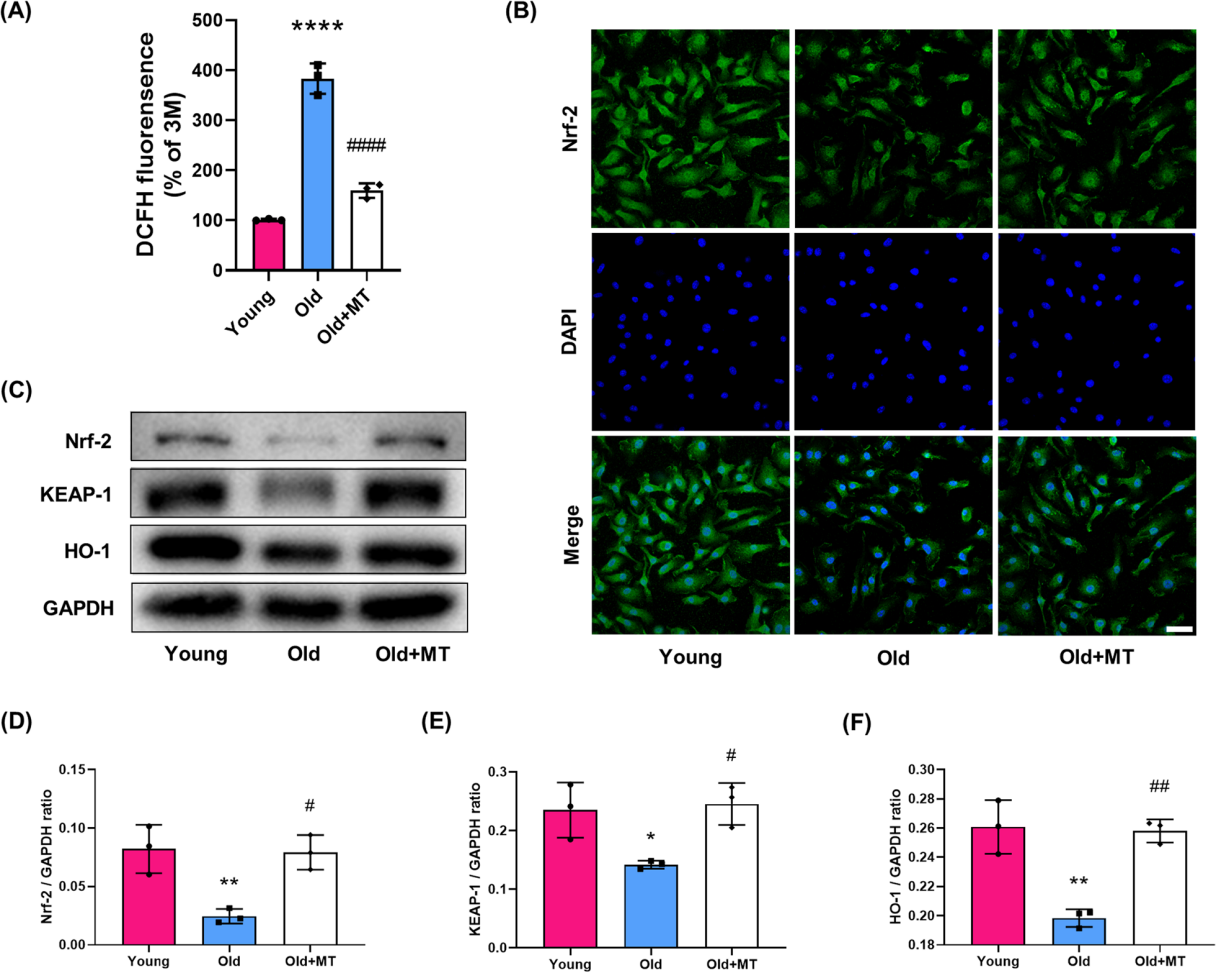
MT enhanced the antioxidant capacity of BMMs from aged mice. **A** DCFH fluorensence analyses of intracellular ROS level in BMMs from young mice, old mice and old mice with MT treatment. Data represent mean ± S.D. of at least three independent experiments (n = 3 per group). **B** Representative fluorescence images of Nrf2 expression. **C**–**F** Western blot analysis of protein levels of Nrf2, Keap1 and HO-1. *p < 0.05, **p < 0.01, ****p < 0.0001 compared with the young group; ^#^p < 0.05, ^##^p < 0.01, ^####^p < 0.0001 compared with the old group

ROS accumulation and oxidative stress contribute to enhanced osteoclastogenesis (Lee et al. 2005). As shown in Fig. 5A, BMMs from aged mice had higher intracellular ROS level than BMMs from young mice, but MT treatment significantly decreased the intracellular ROS level. Under excessive ROS accumulation conditions, some antioxidant enzymes are activated to resist oxidative stress. Keap1/ nuclear factor erythroid 2‐related factor 2 (Nrf2) pathway has been regarded as the main signaling cascade regulating the activation of antioxidant enzymes, including heme oxygenase (HO)-1 (Kanzaki et al. 2017; Hyeon et al. 2013). MT treatment significantly increased the expression of Nrf2, Keap1 and HO-1 in BMMs of aged mice (Fig. 5B–F), which indicates that MT enhanced the antioxidant capacity of BMMs.

### MT supplement attenuated oxidative stress and osteoclastogenesis in aged mice

**Fig. 6:**
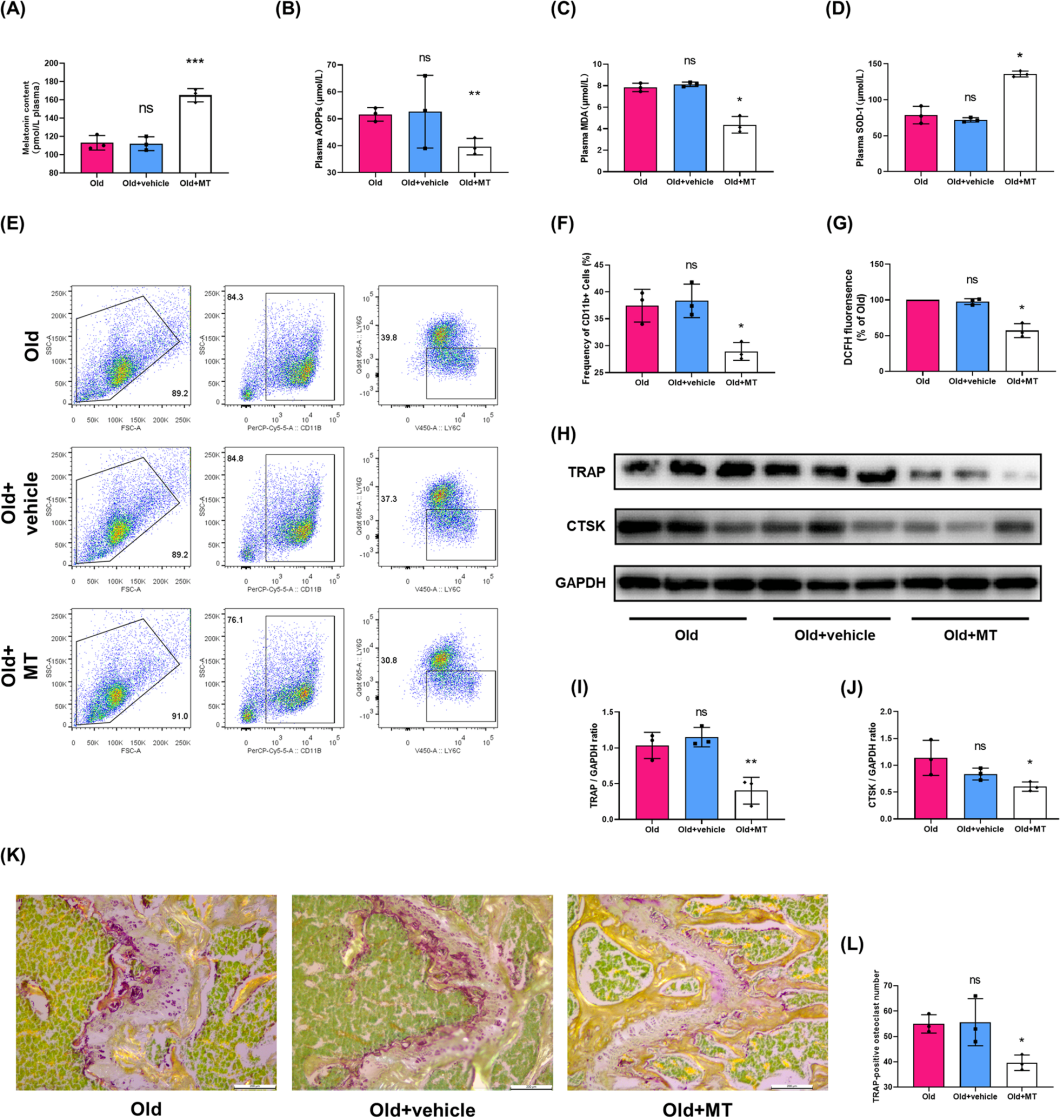
MT supplementation attenuated oxidative stress and osteoclastagenesis in aged mice. **A**–**D** The plasma concentration of MT, AOPPs, MDA and SOD-1 in old mice, old mice with vehicle and old mice with MT treatment. **E**, **F** Flow cytometry analyses of the number and proportion of monocytes in the bone marrow cavity in three groups. **G** DCFH fluorescence analyses of intracellular ROS level in bone marrow monocytes (BMMs) from three groups. Data represent mean ± S.D. of at least three independent experiments (n = 3 per/group). **H**–**J** Western blot analysis of protein levels of TRAP and CTSK in the femoral metaphysis. **K**, **L** Representative images of TRAP staining for assessment of the number of TRAP-positive cells in the femoral metaphysis of old mice, old mice with vehicle and old mice with MT treatment. Scale bar = 200 μm. *p < 0.05, **p < 0.01, ***p < 0.001; ns, not significant

**Fig. 7:**
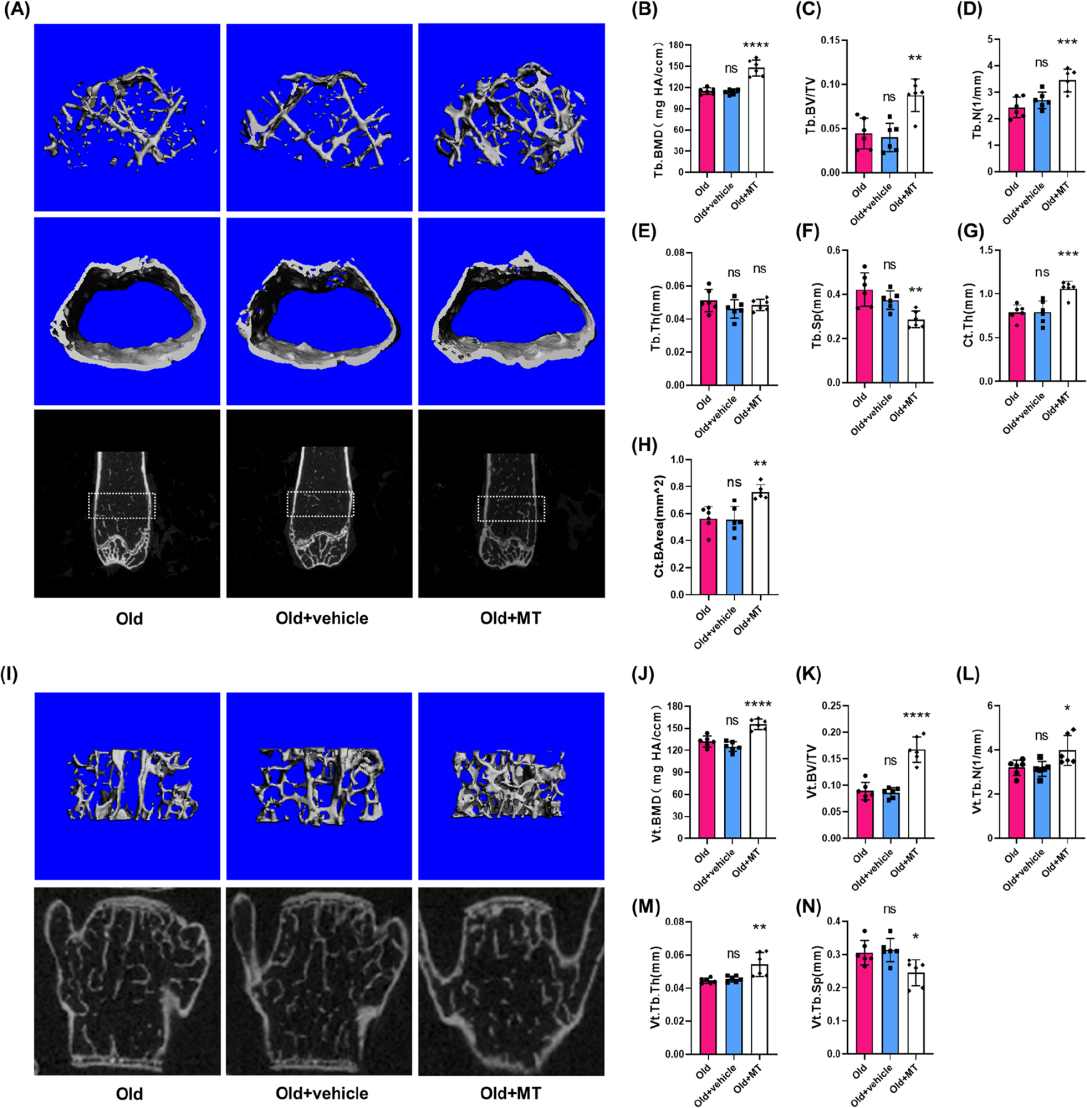
MT supplementation alleviated the deterioration of bone microstructure and bone loss in aged mice. **A** Representative micro-CT images of the distal femur from old mice, old mice with vehicle and old mice with MT treatment. **B**–**H** Quantitative parameters in the distal femur, including bone mineral density (BMD), bone volume fraction (BV/TV), trabecular thickness (Tb/Vt.Th), trabecular number (Tb/Vt.N), trabecular separation (Tb/Vt.Sp), cortical bone thickness (Ct.th) and average cortical bone area (Ct.BArea). **I** Representative μCT images of L4 vertebral bodies. **J**–**N** Quantitative parameters in L4 vertebral bodies. Data represent mean ± S.D. of at least three independent experiments (n = 6 per group). *p < 0.05, **p < 0.01, ***p < 0.001, ****p < 0.0001; ns, not significant

To further determine the effects of MT on osteoclastogenesis in vivo, aged mice were intraperitoneally injected with MT (50 mg/kg/day) for 2 months. As shown in Fig. 6A, the MT supplementation significantly increased the plasma MT level in aged mice. As shown in Fig. 6B–D, the plasma oxidative stress markers, such as AOPPs and MDA, were decreased and SOD-1 was increased after exogenous MT treatment. BMMs also were isolated from MT treated mice and used to analyze the intracellular ROS level. As shown in Fig. 6G, there was a lower intracellular ROS level in BMMs from aged mice with MT treatment compared with aged mice. Furthermore, when aged mice received MT, the number of BMMs in the bone marrow cavity was markedly decreased (Fig. 6E, F). The expression of TRAP and CTSK (Fig. 6H–J) as well as TRAP positive cells (Fig. 6K, L) in metaphysis of femur were also decreased in the MT treated group. We then evaluated the effect of MT on bone microstructure and bone mass. The μCT evaluation showed that MT treatment alleviated bone loss, deterioration of bone microstructure and the thinning of cortical bone in aged mice (Fig. 7). These results suggest that MT treatment attenuated oxidative stress, and suppressed osteoclastogenesis and bone loss in aged mice.

## Discussion

Age-related bone loss increases susceptibility to osteoporosis and fragility fractures, compromising the quality of life in the elderly (Wan et al. 2021; Saul et al. 2022). Although anti-resorptive and anabolic drugs have been developed to address this condition, there are growing concerns about side effects and patient acceptance (Wei et al. 2020). Hence, alternative strategies may be necessary to prevent and treat age-related bone loss. MT is a neurohormone secreted by the pineal gland and known not only as regulator of the body’s circadian rhythm but also as endogenous antioxidant (Galano et al. 2018; Rodriguez et al. 2004; Veneroso et al. 2009; Peyrot et al. 2008; Deng et al. 2006). Exogenous MT may alleviate the pathological process of osteoporosis by reducing oxidative stress and ROS production. Melatonin could antagonize adverse skeletal effects of oxidative stress through scavenging reactive nitrogen species (Oktem et al. 2006). Additionally, melatonin restores the osteogenic potential of bone marrow mesenchymal stem cells lost under conditions like osteoporosis through inhibition of H2O2-induced senescence (Chen et al. 2022). Furthermore, Melatonin improves trabecular microstructure in retinoic acid-induced osteoporosis mice by reducing oxidative stress through ERK/SMAD and NF-κB pathways (Wang et al. 2019). Besides the reported potential antioxidant effects of melatonin, research suggests that the serum MT level declines considerably with aging and contributes to the development of many age-related diseases (Claustrat et al. 2005), which underscores the importance of our findings. In this study, we demonstrated that MT level is linked with the changes in osteoclastogenesis and bone mass in the aging process, and MT supplementation can inhibit osteoclastogenesis and improve bone loss by enhanced antioxidant capacity. Therefore, MT as an antioxidant may be a promising candidate for preventing or alleviating age-related bone loss.

Enhanced osteoclastogenesis is one of the major causes of age-related bone loss (Cao et al. 2005). The number of osteoclast progenitors increases with aging (Perkins et al. 1994), and these osteoclast progenitors show an increased ability to differentiate into mature osteoclasts and bone resorption (D'Amelio et al. 2005; Ziegler-Heitbrock. 2014). In this study, we found that aged mice showed an increased number of BMMs in the bone marrow cavity, higher capacity of osteoclastogenesis and lower bone mass compared with young mice. It was reported that MT can significantly inhibit osteoclast differentiation via attenuating intracellular ROS level in a dose-dependent manner at pharmacological concentrations (1–100 μM), but not at physiological concentrations (0.01–10 nM) (Zhou et al. 2017). Moreover, intraperitoneal injection of MT can prevent bone loss in mice with type 1 diabetes mellitus (Gong et al. 2022). This current study presented evidence that age-related decline of MT is associated with enhanced osteoclastogenesis and bone loss, and the MT challenge markedly suppresses osteoclastogenesis in vitro. Furthermore, MT supplementation exhibited a decrease of BMMs in the bone marrow cavity, and alleviated bone loss and deterioration of bone microstructure in aged mice. Therefore, MT plays an important role in bone homeostasis via the suppression of osteoclastogenesis and bone resorption activity.

Disruption of redox homeostasis may lead to oxidative stress and is involved in the aging process (Liguori et al. 2018; Balaban et al. 2005). With aging, excessive oxidative stress has extensive negative effects on bone remodeling, thereby inducing bone loss as well as deterioration of bone quality and mechanical strength (Domazetovic et al. 2017). ROS act as intracellular signaling molecules involved in osteoclastogenesis. Doxorubicin-induced bone loss is through a massive accumulation of ROS and nitrogen species leading to oxidative stress (Poudel et al. 2022). Moreover, Metastasis-associated protein 1 deficiency inhibited Nrf2 nuclear translocation and Increased intracellular ROS levels, leading to enhanced osteoclast formation (Feng et al. 2023). Accumulation of ROS is involved in enhanced osteoclastogenesis and bone resorption, leading to an imbalance in skeletal turnover and bone loss (Baek et al. 2010). In this study, elevated oxidative stress, reflected in increased plasma AOPPs and MDA levels, was observed in aged mice. Moreover, the intracellular ROS level was significantly higher in BMMs from aged mice than that in young mice. MT supplementation can markedly reverse osteoclastogenesis and bone loss by alleviating oxidative stress. Therefore, MT influences osteoclastogenesis via regulating redox homeostasis.

The present study had several limitations. First, age-related bone loss, a highly complex process, was investigated only in young and old mice. This model may not fully reflect its complexities in humans. Second, we focus on the impact of MT on osteoclastogenesis but not on osteogenesis. Actually, bone loss results from an imbalance between osteoblastic bone formation and osteoclastic bone resorption in the natural aging process (Sfeir et al. 2022). Previous study has demonstrated that MT can enhance osteogenic differentiation of bone marrow mesenchymal stem cells and restore oxidative stress-inhibited osteogenesis (Lee et al. 2018). In our study, MT treatment may improve bone mass in old mice in part by restoring osteogenesis and bone formation. Furthermore, circadian rhythm disruption easily leads to bone loss and osteoporosis (Swanson et al. 2017). Evidence has shown that the circadian system may be a promising intervention for the treatment of abnormal bone metabolism (Song et al. 2018). It is necessary to further clarify the effect of MT on age-related bone loss by regulating the body’s circadian rhythm.

## Conclusions

Our results suggest that MT level declines with aging, which is associated with disruption of redox homeostasis. Age-related decline of MT contributes to enhanced osteoclastogenesis and bone loss. Exogenous MT supplementation can attenuate osteoclastogenesis and improve bone loss through enhancing antioxidant capacity. Collectively, MT as a dietary supplementation and/or medicinal drug is beneficial in the prevention and treatment of age-related bone loss and senile osteoporosis.

### Supplementary Information

**Additional file 1: Figure S1.** The effects of different concentrations of MT on the osteoclastogenesis of BMMs. (A) Representative TRAP staining images of osteoclast differentiation of BMMs in vitro after treatment of MT at different concentrations. (B) Representative rhodamine's Phalloidin staining for F-actin ring formation in BMMs. (C) Cell proliferation was quantified at separate time points of 1, 2, 3, 4, 5 and 6 d and showed no significant differences between MT treated and untreated groups of BMMs. (D) DCFH fluorescence analyses of intracellular ROS level in BMMs after treatment of MT at different concentrations. Data represent mean ± S.D. of at least three independent experiments (n = 3 per/group). *p < 0.05, **p < 0.01.

## Data Availability

The data and materials of the study can be obtained from the corresponding author upon request.
