# Supplementary figures and images for: Age-related decline in melatonin contributes to enhanced osteoclastogenesis via disruption of redox homeostasis
Source: Mol Med. 2024 Jan 12;30:10. doi: 10.1186/s10020-024-00779-x (PMC10785421; doi:10.1186/s10020-024-00779-x)

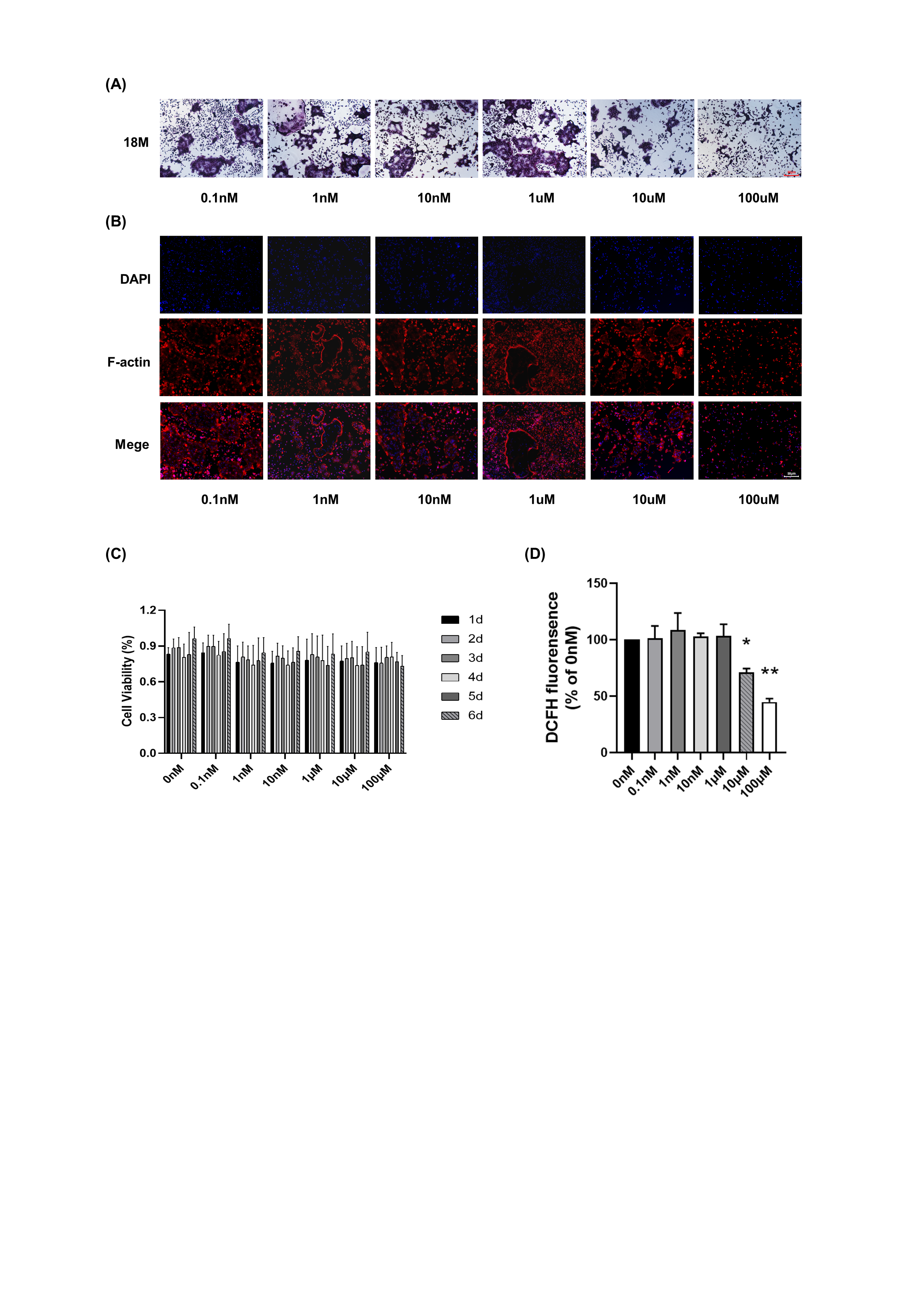

Supplement: Supplementary file 1 — Additional file 1: Figure S1. The effects of different concentrations of MT on the osteoclastogenesis of BMMs. (A) Representative TRAP staining images of osteoclast differentiation of BMMs in vitro after treatment of MT at different concentrations. (B) Representative rhodamine's Phalloidin staining for F-actin ring formation in BMMs. (C) Cell proliferation was quantified at separate time points of 1, 2, 3, 4, 5 and 6 d and showed no significant differences between MT treated and untreated groups of BMMs. (D) DCFH fluorescence analyses of intracellular ROS level in BMMs after treatment of MT at different concentrations. Data represent mean ± S.D. of at least three independent experiments (n = 3 per/group). *p < 0.05, **p < 0.01. [file 10020_2024_779_MOESM1_ESM.tif]
